# LAIR-1 overexpression inhibits epithelial–mesenchymal transition in osteosarcoma via GLUT1-related energy metabolism

**DOI:** 10.1186/s12957-020-01896-7

**Published:** 2020-06-20

**Authors:** Jinxue Zhang, Yuan Zhang, Shiyang Cheng, Yang Mu, Yongming Liu, Xin Yi, Dongxu Jiang, Yong Ding, Ran Zhuang

**Affiliations:** 1 Orthopedic Department of Tangdu Hospital, Fourth Military Medical University, #1 Xinsi Road, Xi’an, 710032 China; 2 Department of Immunology, Fourth Military Medical University, #129 West Changle Road, Xi’an, 710032 China; 3 Institute of Medical Research, Northwestern Polytechnical University, 127 West Youyi Road, Xi’an, 710072 Shaanxi China

**Keywords:** Osteosarcoma, Leukocyte-associated immunoglobulin-like receptor-1, Epithelial–mesenchymal transition, Glucose transporter 1

## Abstract

**Background:**

Leukocyte-associated immunoglobulin-like receptor-1 (LAIR-1) is a collagen receptor belonging to the immunoglobulin superfamily. Although previous studies have evaluated the biological role of LAIR in solid tumors, the precise mechanisms underlying the functions of LAIR-1 as a regulator of tumor biological functions remain unclear.

**Methods:**

LAIR-1 expression was evaluated by immunohistochemical analysis using an osteosarcoma (OS) tissue microarray. Wound healing and transwell migration assays were performed to evaluate tumor cell migration. Quantitative real-time polymerase chain reaction (qPCR) and western blotting were conducted to detect the expression of epithelial–mesenchymal transition (EMT)-related molecules. RNA-sequencing (RNA-seq) was conducted to evaluate the mRNA expression profiles after overexpressing LAIR-1 in OS cells. Glucose transporter (Glut)1 expression in OS cells was evaluated by western blotting.

**Results:**

LAIR-1 expression was significantly different between the T1 and T2 stages of OS tumors, and it inhibited OS cell migration. LAIR-1 expression was inversely correlated with the expression of Twist1, an EMT-associated transcription factor, via the Forkhead box O1 signal transduction pathway. Furthermore, RNA-seq and qPCR demonstrated that the expression of EMT energy metabolism-related molecules was significantly reduced after LAIR-1 overexpression.

**Conclusions:**

LAIR-1 overexpression decreased the expression of Glut1 and inhibited the expression of EMT-related molecules in OS cells. These findings provide new insights into the molecular mechanism underlying OS progression.

## Background

Osteosarcoma (OS) is the most common malignant solid bone tumor in children and young adults, accounting for 6% of all pediatric cancers and typically originating in the metaphyses of long bones [1, 2]. Multidisciplinary approaches have been developed for treating patients with OS [3, 4]; however, their overall prognosis remains unsatisfactory, with a 5-year survival rate as low as 20% [5–7]. Therefore, new and effective targets for OS diagnosis, therapy, and prognosis are warranted.

Leukocyte-associated immunoglobulin-like receptor-1 (LAIR-1; also known as CD305) is a collagen receptor and member of the immunoglobulin superfamily [8]. Studies evaluating the functions of LAIR-1 have mainly focused on immune cells, such as T cells, B cells, natural killer cells, monocytes, megakaryocytes, and CD34^+^ hematopoietic progenitor cells [8, 9]. LAIR-1 has been indicated to play important roles in different types of cancers, including leukemia and solid tumors [10–14].

Against this background, the present study aimed to evaluate the role of LAIR-1 in OS progression by determining LAIR-1 expression in OS tissues. We overexpressed LAIR-1 in OS cell lines by lentiviral transfection and measured cell proliferation, epithelial–mesenchymal transition (EMT)-associated transcription factor expression, and cell migration. Furthermore, EMT-related energy metabolism was examined after LAIR-1 overexpression. We believe that our study provides novel insights into the role of LAIR-1 in OS.

## Materials and methods

### Immunohistochemistry

We used a formalin-fixed paraffin-embedded tissue microarray comprising 62 samples from patients with OS and 9 samples from adjacent normal rib bone tissues (Alenabio Biological Technology Company; Xi’an, Shaanxi, China). Clinicopathological data, including age, sex, pathological diagnosis, tumor–node–metastasis grading, and tumor stage, were collected from the medical records of surgically treated patients with OS. No patients had been administered preoperative treatment or had the co-occurrence of other diagnosed tumors. The sample size of patients with T3 stage OS tumor was small (*n* = 3), and thus, this group was excluded from the analysis. Samples for IHC analysis were prepared using a standard method.

### Semiquantitative analysis of immunohistochemical data and bioinformatics analysis

All tissue samples were evaluated by two independent pathologists blinded to the clinical data. A semiquantitative score was generated based on the IHC staining intensity as follows: +, weak staining; ++, moderate staining; and +++, intense staining. The R2 platform (http://r2.amc.nl) was used to analyze the public OS dataset, which includes 127 OS samples.

### Cell culture

Human OS cell lines were purchased from ATCC (Manassas, VA, USA), and the human normal osteoblast cell line hFOB1.19 was obtained from Jennio Biotech (Guangzhou, Guangdong, China). All cells were cultured at 37 °C in a humidified atmosphere comprising 5% CO_2_. Foxo1 short interfering RNA (siRNA; sc-35382) and negative control (NC) siRNA were purchased from Santa Cruz Biotechnology (Dallas, TX, USA). Cells were transfected with 50 nM Foxo1 siRNA or NC siRNA using Lipofectamine 3000 (Invitrogen, Carlsbad, CA, USA), according to the manufacturer’s instructions.

### Lentivirus infection

Commercially available lentiviral (LV)-LAIR-1 constructs (Tianyucheng Biotechnology, Xi’an, Shaanxi, China) were modified to overexpress LAIR-1. Human OS cells were infected with LV-NC or LV-LAIR-1. The infection efficiency of LV vectors expressing green fluorescent protein (GFP) was evaluated by fluorescence microscopy.

### Quantitative real-time polymerase chain reaction

Total RNA was extracted from cells using TRIzol Reagent (Invitrogen). SuperScript III Reverse Transcriptase (Invitrogen) was used for reverse transcription, and PCR was performed using the SYBR Green Realtime PCR Master Mix (TAKARA, Shiga, Japan). Quantitative real-time polymerase chain reaction (qPCR) primers for human genes were purchased from Tsingke Biotech (Beijing, China). Melting curve analysis was performed to verify the specificity of the primers. Relative gene expression was quantified using the comparative Ct method (2^−ΔΔCT^), with glyceraldehyde 3-phosphate dehydrogenase (GAPDH) used as an internal control.

### Western blotting analysis

Total protein was extracted using a routine procedure and blotted with the following primary antibodies: LAIR-1 (sc-398141; Santa Cruz Biotechnology), phospho-Foxo1 (Ser256) (84192; Cell Signaling Technology, Danvers, MA, USA), Foxo1 (2880; Cell Signaling Technology), phospho-Akt (Ser473) (AF8355; Affinity Biosciences, Cincinnati, OH, USA), Akt (9272; Cell Signaling Technology), proliferating cell nuclear antigen (PCNA; BM0104; Boster Biotech Co., Ltd., Wuhan, China), Twist1 (ab50581; Abcam, Cambridge, UK), Glut1 (NB110-39113, Novus Biologicals, Littleton, CO, USA), and β-actin (30101ES50; Yeasen Biotech Co., Ltd., Shanghai, China).

### Wound healing and transwell migration assays

Cells were seeded into six-well plates, and a scratch was produced in the monolayer after 48 h. Images of the wounded region were captured immediately after the scratch and after 6 and 12 h (T0, T6, and T12, respectively) to monitor cell migration into this region. The percentage of the scratch area (% scratch) that showed wound closure was calculated as follows:

% scratch = (width at T0 − width at T6 or T12)/width at T0 × 100

Transwell migration assay was performed using a transwell chamber with 8-μm pores (Millipore, Billerica, MA, USA). Untreated OS cells (blank group) and their corresponding transfectants overexpressing LV-NC and LV-LAIR-1 were seeded (2 × 10^4^ into each well) into the upper chambers in 500 μL of serum-free medium. The lower chambers were filled with complete medium, and all chambers were incubated at 37 °C for 24 h. The cells on the upper surface of the membrane were removed, whereas those in the lower chamber were fixed and stained with 0.1% crystal violet. Images were obtained using an inverted microscope (CX41, Olympus, Tokyo, Japan).

### Immunofluorescent staining

Cells were cultured on glass chamber slides. OS cells were fixed, permeabilized, and incubated for 1 h at room temperature with the following primary antibodies: Glut1 (NB110-39113, Novus Biologicals, Littleton, CO, USA), Twist1 (ab50581; Abcam, Cambridge, UK), and Foxo1 (2880; Cell Signaling Technology). After washing, the cells were incubated with Cy3-labeled goat anti-rabbit secondary antibody and stained with 4′,6-diamidino-2-phenylindole (DAPI) (Roche Diagnostics, Basel, Switzerland). Images were obtained using an Olympus microscope. Immunofluorescence intensities were quantified using the ImageJ software (NIH, Bethesda, MD, USA).

### RNA-sequencing

Total RNA was extracted from HOS cells transfected with LV-NC (*n* = 3) and LV-LAIR-1 (*n* = 3) using an RNeasy Micro kit (Qiagen, Hilden, Germany), according to the manufacturer’s instructions. RNA was quantified using a NanoDrop ND-2000 spectrophotometer (Nanodrop Technologies, Inc., Wilmington, DE, USA). Generation of the transcriptome library and RNA-sequencing (RNA-seq) was performed on the BGISEQ platform (BGI Technologies, Shenzhen, China). The Dr. TOM2 system (BGI Technologies) was used to analyze the transcriptome data.

### Statistical analysis

Data were statistically analyzed using the GraphPad Prism version 6.0 software (GraphPad, Inc., La Jolla, CA, USA); all data are presented as mean ± standard deviation. Data were analyzed using an independent sample *t* test for comparisons between two groups. Clinical data were statistically analyzed using the SPSS software (version 10.0; SPSS, Inc., Chicago, IL, USA). Pearson *χ*^2^ test was used to evaluate the statistical significance of the association between LAIR-1 expression and clinical data (*n* = 59). *P* values of < 0.05 were considered statistically significant.

## Results

### Increased LAIR-1 expression is associated with advanced T stage in patients with OS

**Fig. 1:**
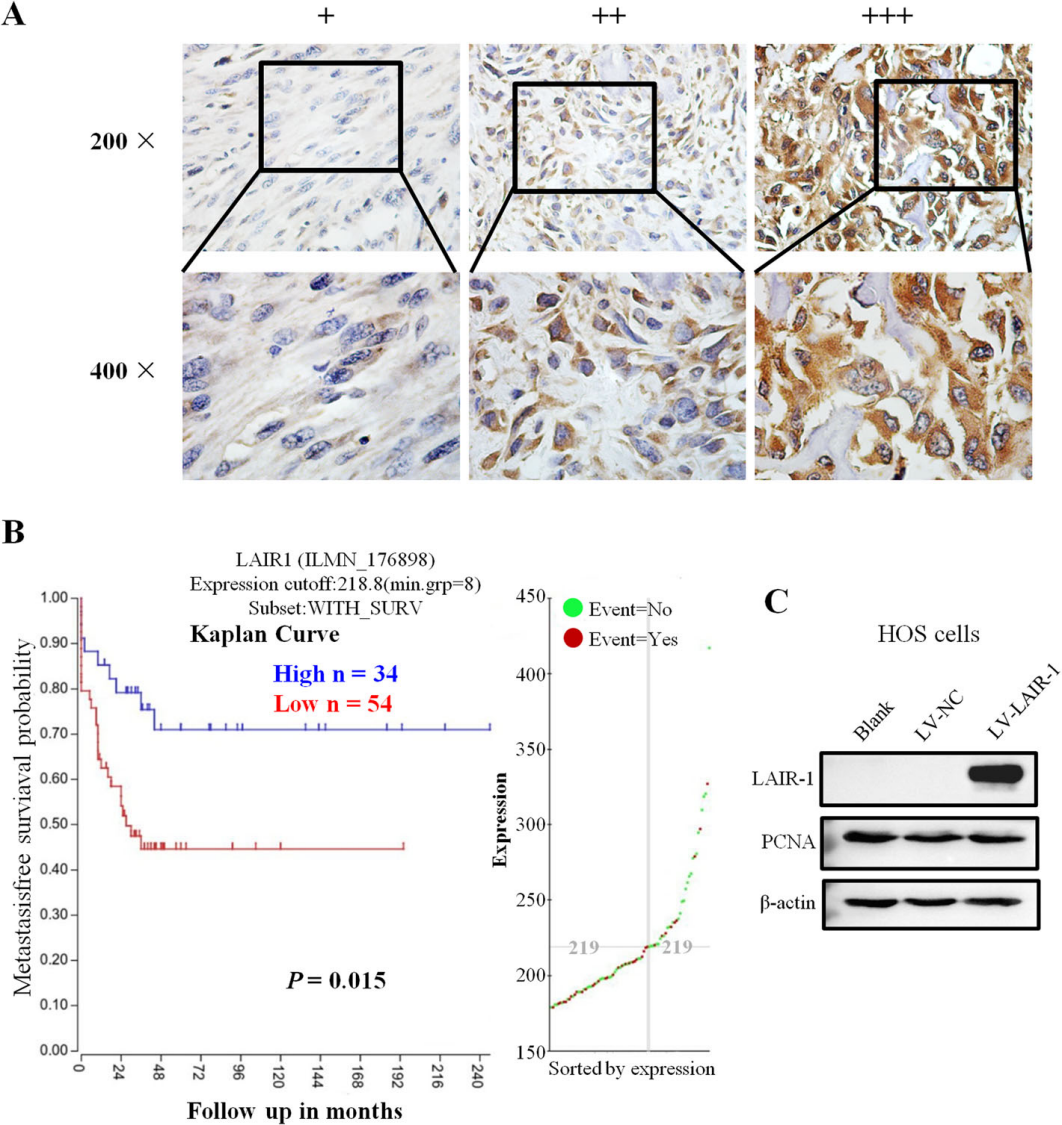
Representative images of different LAIR-1 immunohistochemistry staining intensities in OS tissues. The proportion of positively stained cells for LAIR-1 was calculated by assessing the entire image. Based on the LAIR-1 staining intensities in OS tumor samples, the staining patterns were categorized as follows: weak (+), moderate (++), and intense (+++). Upper panel, original magnification × 200; lower panel, original magnification × 400. **b** Kaplan–Meier plot of survival rates of patients with tumors exhibiting high (blue line) or low (red line) LAIR-1 expression; data were obtained using the R2 platform. **c** Western blotting for determining LAIR-1 expression and PCNA proliferation marker levels in HOS cells following LV-NC or LV-LAIR-1 lentivirus infection or without treatment (blank). β-actin was used as a loading control

IHC staining was performed to detect LAIR-1 expression in 62 human OS samples and 9 adjacent normal bone tissues. Unlike the membrane expression pattern in lymphocytes, which have a large nucleus and a small cytoplasmic volume, we observed a higher LAIR-1 expression in the cytoplasm of OS cells than in the cell membrane (Fig. 1a). Comparisons between LAIR-1 expression and the clinicopathological characteristics of patients with OS are shown in Table 1. LAIR-1 expression was significantly higher in patients with T2 stage OS tumor than in those with T1 stage OS tumor (*P* = 0.006).

**Table 1:** Relationship between LAIR-1 expression and clinicopathological features in OS patients (*n* = 59)

Variable		No. of patients	LAIR-1 expression			*P* values
			+	++	+++	
Gender	Male	37 (62.7%)	7	14	16	0.871
	Female	22 (37.3%)	3	9	10	
Age (years)	> 20	38 (64.4%)	3	16	19	**0.037**
	< 20	21 (35.6%)	7	5	9	
T stage	T1	17 (28.8%)	7	5	5	**0.006**
	T2	42 (71.2%)	3	16	23	

*P* values based on χ^2^ test; bold, statistically significant (*P* < 0.05)

Moreover, analysis of 127 OS samples using the R2 platform indicated that patients with higher LAIR-1 expression levels had better survival rates than those with lower LAIR-1 expression levels (*P* = 0.015, Fig. 1b). According to these observations, we investigated the association between LAIR-1 overexpression and human OS tumor growth and examined whether LAIR-1 overexpression exhibits a compensatory effect to help overcome OS progression.

### LAIR-1 overexpression inhibited OS cell migration

We compared the LAIR-1 expression levels in different human OS cell lines and the human osteoblast cell line hFOB1.19 (Supplementary Fig. 1A). To investigate the biological function of LAIR-1 overexpression in OS pathogenesis, we designed a recombinant lentivirus to overexpress LAIR-1 in OS cell lines with low LAIR-1 expression levels (i.e., HOS cell line). The GFP-positive cell ratio determined in these cells by fluorescence microscopy was > 95% (Supplementary Fig. 1C), demonstrating that the LV-LAIR-1 lentivirus had a high transduction efficiency for HOS cells. LAIR-1 overexpression at the mRNA and protein levels in HOS cells was confirmed by qPCR (Supplementary Fig. 1D) and western blotting (Fig. 1c), respectively.

In addition, western blotting revealed no differences in the expression of PCNA, a protein marker related to cell proliferation, in all the groups (Fig. 1c). EdU cell proliferation assay and western blotting were performed to determine PCNA expression to analyze whether LAIR-1 overexpression affects the growth and proliferation of human OS cells. The EdU assay revealed no difference in the number of positively stained HOS cells between the LAIR-1 overexpression group and LV-NC or blank groups (Supplementary Fig. 1E). These findings demonstrated that LAIR-1 overexpression did not affect OS cell growth or proliferation.

**Fig. 2:**
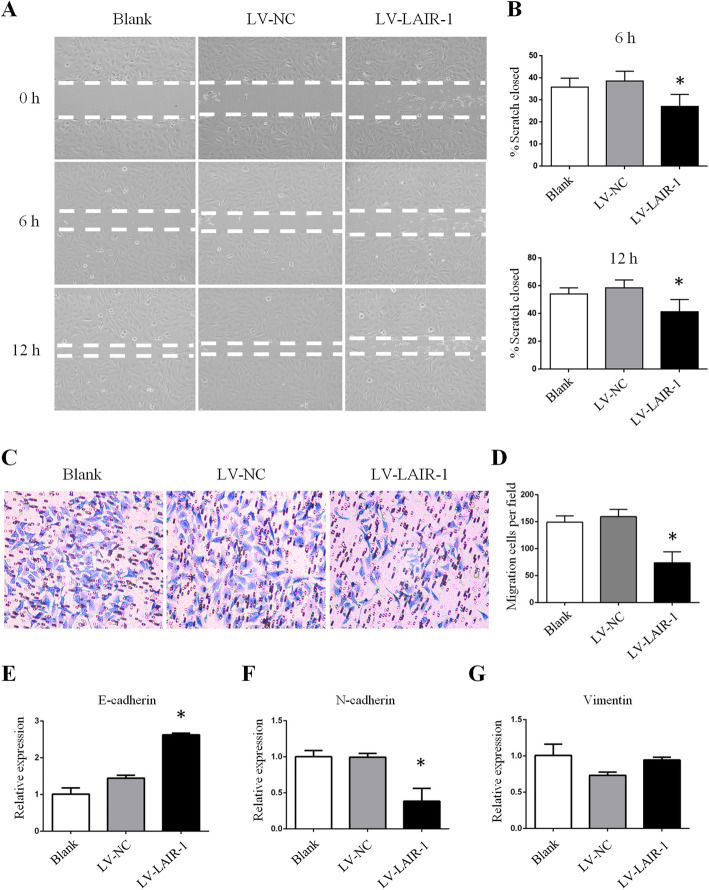
LAIR-1 overexpression inhibits HOS cell migration. **a** Representative images of cell migration for wound closure. **b** Statistical ratio of wound closure after scratch formation. **c** Representative images of transwell membranes stained with crystal violet showing a decreased number of cells after LAIR-1 overexpression. **d** Relative ratio of migratory cells per field. e–**g** LAIR-1 overexpression changes the mRNA expression levels of EMT markers in HOS cells, as determined by qPCR. * *P* < 0.05

Next, we investigated the effect of LAIR-1 expression on OS metastasis in wound healing and transwell migration assays. The scratch assay showed significantly lower repair efficiency in LAIR-1-overexpressing OS cells than in LV-NC-overexpressing and untreated blank cells (Fig. 2a). The results of the statistical analysis of the scratch closure ratios at 6 and 12 h are shown in Fig. 2b. We also conducted a cell migration assay to analyze the migration ability of OS cells after ectopic LAIR-1 overexpression. The results indicated that the migration ability of OS cells treated with LV-LAIR-1 was drastically lower than that of untreated blank cells and cells treated with LV-NC (Fig. 2c, d). These results demonstrated that LAIR-1 overexpression inhibits OS cell migration.

### LAIR-1 overexpression suppressed EMT in OS cells

We further explored the mechanism underlying the suppression of OS cell migration by LAIR-1 overexpression. qPCR revealed that compared with that observed in untreated blank cells and cells treated with LV-NC, the E-cadherin mRNA expression was upregulated and the N-cadherin mRNA expression was downregulated in LAIR-1-overexpressing OS cells (Fig. 2e, f); however, no change was observed in the vimentin mRNA expression in LAIR-1-overexpressing OS cells (Fig. 2g).

**Fig. 3:**
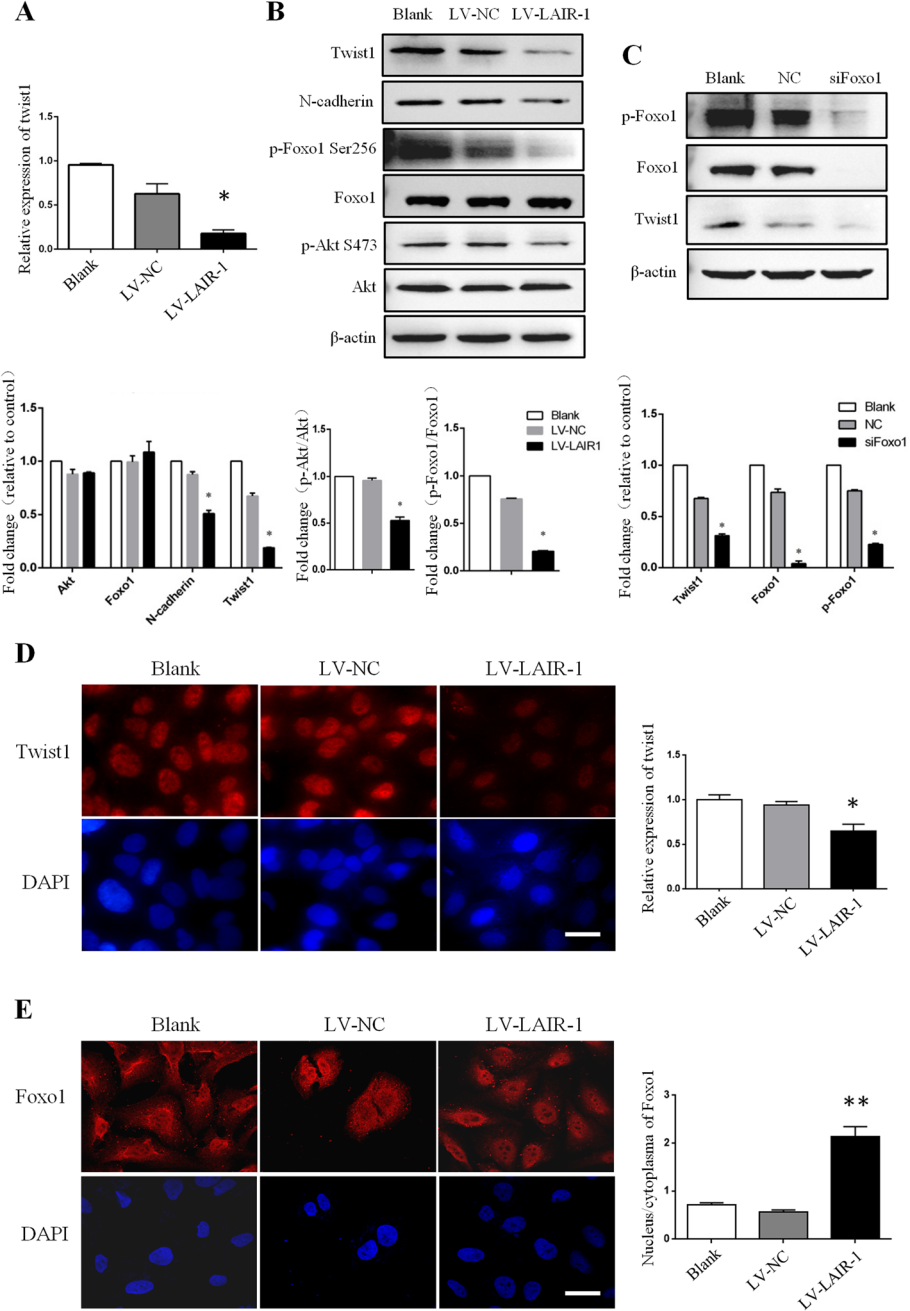
LAIR-1 overexpression in HOS cells inhibits the expression of EMT-associated transcription factors by decreasing p-Foxo1 expression. **a** Expression of *Twist1* in untreated HOS cells (Blank) and corresponding LV-NC- and LV-LAIR-1-overexpressing transfectants was analyzed by qPCR. **b** Twist1, N-cadherin, p-Foxo1, total Foxo1, p-Akt, and total Akt expression were analyzed by western blotting in blank, LV-NC-overexpressing, and LV-LAIR-1-overexpressing OS cells. **c** Twist1, p-Foxo1, and total Foxo1 expression were analyzed by western blotting in blank, negative control (NC) siRNA, and Foxo1 siRNA (siFoxo1)-transfected OS cells. Representative immunofluorescence staining for Twist1 (**d**) and Foxo1 (**e**) in HOS cells (left). Cell nuclei were stained with DAPI (blue). Scale bar = 20 μm. The statistical data for the images are shown in histograms (right). *P < 0.05, **P < 0.01. Results represent at least three independent experiments.

Here LAIR-1 overexpression significantly decreased the mRNA and protein levels of Twist1 in HOS cells compared with that observed in the untreated blank cells and cells treated with LV-NC (Fig. 3a, b). In addition, immunofluorescence staining revealed that Twist1 expression was significantly downregulated after LAIR-1 overexpression in HOS cells (Fig. 3d).

Evaluation of the Foxo1 expression levels in OS cells showed that LAIR-1 overexpression decreased Foxo1 phosphorylation. Immunofluorescence staining further demonstrated increased nuclear retention of Foxo1 (Fig. 3b, e). Phosphorylation of Akt, the direct upstream regulator of Foxo1, was markedly decreased in LAIR-1-overexpressing OS cells, indicating that decreased Foxo1 phosphorylation and increased Foxo1 retention in the nucleus are regulated by decreased Akt activation (Fig. 3b).

The role of Foxo1 in decreasing Twist1 expression was further confirmed in OS cells. Twist1 expression was decreased in HOS cells transfected with Foxo1 siRNA compared with that observed in cells transfected with NC siRNA (Fig. 3c). These results suggested that LAIR-1 overexpression significantly decreases the expression of EMT-related molecules in OS cells via Twist1, which functions downstream of phosphorylated Foxo1.

### Characterization of EMT-related genes in LAIR-1-overexpressing OS cells

**Fig. 4:**
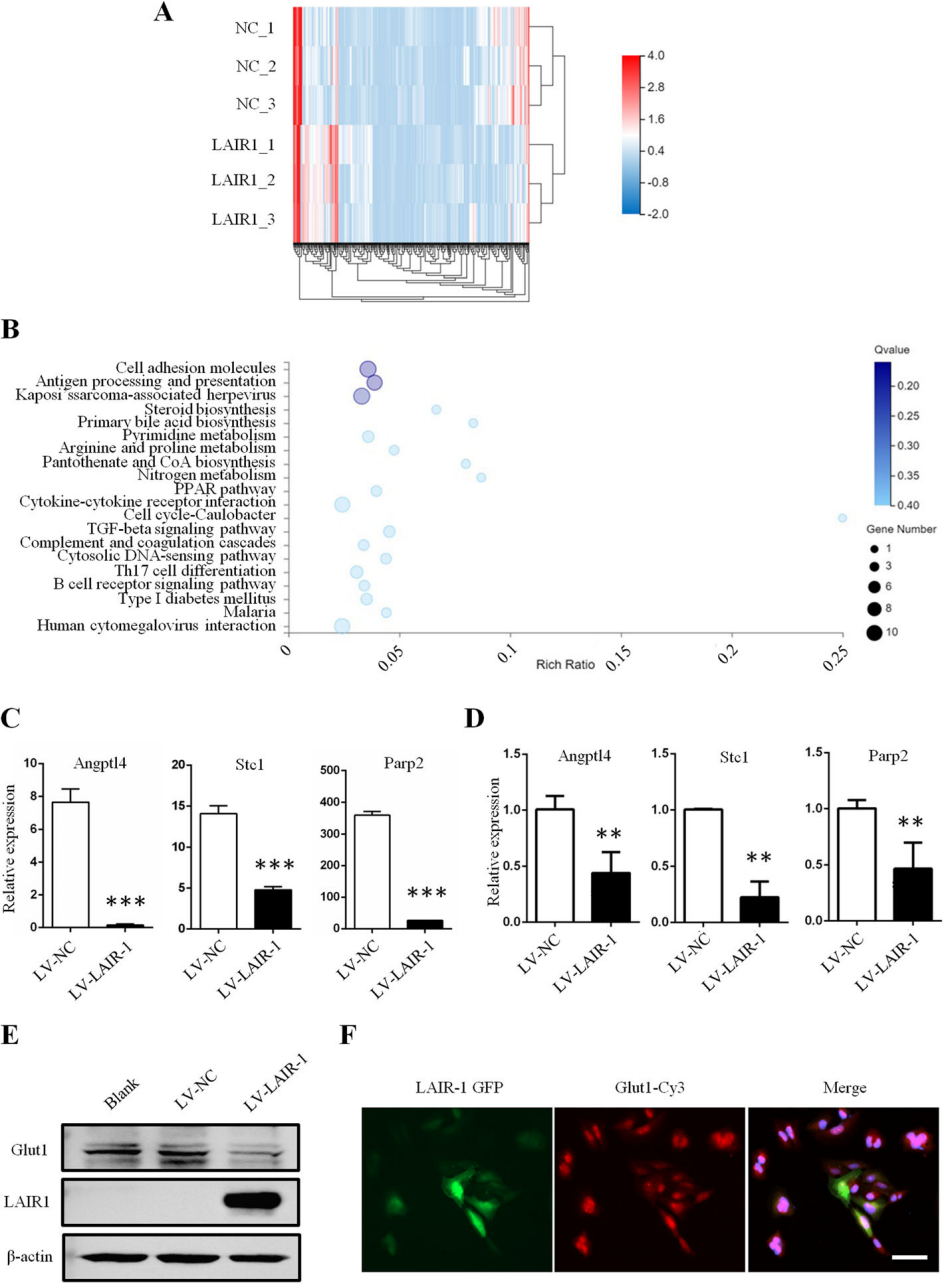
LAIR-1 inhibits Glut1-related glucose uptake in OS cells. **a** Heatmap showing the levels of differentially expressed mRNAs. **b** Top 20 KEGG pathway annotation categories for target gene functions of predicted mRNAs. **c** Selected significantly differentially expressed mRNA-related to EMT in RNA-seq data between two groups, ***P < 0.001. **d** qPCR validation of differentially expressed EMT-related genes in LV-NC and LV-LAIR-1-overexpressing OS cells, **P < 0.01. **e** Glut1 expression analyzed by western blotting. **f** Immunofluorescence staining of Glut1 in the LV-LAIR-1-overexpressing OS cells. Scale bar = 50 μm. Data were obtained from at least two independent experiments.

To further clarify the mechanism involved in the LAIR-1 overexpression-mediated decrease in the expression of EMT-related molecules, RNA-seq was performed to detect the mRNA expression profiles of these EMT-related genes in OS cells (reference genome: GCF_000001405.38_GRCh38.p12). We identified that 974 mRNAs expressed specifically in LAIR-1-overexpressing OS cells and 1064 mRNAs expressed specifically in cells treated with LV-NC, as revealed by RNA-seq analysis. Compared with that observed in cells treated with LV-NC, 327 mRNAs showed significantly differential expression in LAIR-1-overexpressing OS cells (173 upregulated and 154 downregulated, log2 fold-change ≥ 1.0 and false discovery rate < 0.05). The RNA-seq analysis details are presented in Supplementary Table 1. A heatmap was generated to show the respective hierarchical clustering of mRNA with altered levels in cells treated with LV-NC and LAIR-1-overexpressing OS cells (Fig. 4a).

Furthermore, Kyoto Encyclopedia of Gene and Genomes (KEGG) pathway analysis of the top 20 enriched mRNAs potentially acting on target genes and regulating OS progression were identified (Fig. 4b). Interestingly, the expression of several mRNAs reported to play key roles in EMT was significantly reduced in LAIR-1-overexpressing OS cells (Fig. 4c); these included angiopoietin-like (ANGPTL) 4, stanniocalcin (STC) 1, and poly ADP-ribose transferase (PARP) 2.

### LAIR-1 inhibits EMT via Glut1-related energy metabolism

The ANGPTL4, STC1, and PARP2 mRNAs differently expressed between the LV-NC and LAIR-1-overexpressing OS cells were validated by qPCR, and the results were consistent with the RNA-seq data (Fig. 4d). EMT is an energy-demanding process fueled by glucose metabolism-derived ATP. Notably, ANGPTL4, STC1, and PARP2 are involved in the energy metabolism associated with EMT. These data suggested that LAIR-1 overexpression inhibits EMT via metabolic-related processes. EMT is accompanied by upregulated glucose consumption, as evidenced by upregulated Glut1 expression [15]. Here we observed a significant decrease in Glut1 expression in LAIR-1-overexpressing OS cells compared with that observed in control cells (Fig. 4e). As shown in Fig. 4f, immunofluorescence staining also demonstrated decreased Glut1 expression in OS cells with highly expressed LAIR-1. These results indicated the involvement of LAIR-1 in EMT in OS via Glut1-related energy metabolism.

## Discussion

The inhibitory receptor LAIR-1 is a member of the immunoglobulin superfamily, and it binds to extracellular matrix collagens, which have been identified as high-affinity ligands for LAIR-1 molecules [16]. Functionally, LAIR-1 tyrosine-based inhibition motifs utilize tyrosine phosphorylation to recruit phosphatases and negatively regulate immune response and cell differentiation [17]. In addition, the interaction between LAIR-1 and collagen can facilitate the binding of tumor cells to inhibitory molecules on immune cells to inhibit antitumor immune responses, suggesting their role in tumor immune evasion [18]. Previous studies have demonstrated the biological function of LAIR-1 in solid tumors, such as ovarian cancer, cervical cancer, and hepatocellular carcinoma [10–12]. Here we found that high LAIR-1 expression was associated with a more advanced OS tumor stage (*P* = 0.006). Moreover, analysis of 127 OS samples using the R2 platform demonstrated that LAIR-1 expression was correlated with the survival rates of patients with OS.

Next, for in vitro experiments, we first detected LAIR-1 expression in five different OS cell lines to determine whether the phenotype and function of LAIR1 is different in these cell lines. Compared with the human normal osteoblast cell line hFOB1.19, most cells of the OS cell lines studied here, except for MG63, showed reduced expression levels of LAIR-1. Several studies have demonstrated that MG63 is an OS cell line with poor tumorigenicity [19]; thus, HOS cells, which have demonstrated high poor tumorigenicity, with the lowest expression level of LAIR1 were used in this study.

Although OS is suspected to originate from dysfunctional mesenchymal stromal/stem cells, studies have demonstrated an association between EMT and OS migration [20]. Interestingly, the collagen matrix can induce EMT in OS cells via extracellular signal-regulated kinase signaling [21]. The expression of LAIR-1, as an important collagen ligand, on tumors may regulate EMT in OS cells. In the present study, we found that LAIR-1 overexpression decreased N-cadherin expression but did not affect vimentin expression. Although the mRNA level of E-cadherin was increased after LAIR-1 overexpression, the protein level was only slightly upregulated. In most tumors, N-cadherin can be used as a trigger of tumor invasion and its expression is often upregulated. E-cadherin demonstrates a more extensive expression than N-cadherin on cells, including those of normal tissues and tumors, suggesting that the regulation of E-cadherin expression is influenced by more complicated mechanisms [22]. Moreover, the in vitro wound healing and transwell migration assays provided evidence that cell motility and migration are remarkably inhibited by LAIR-1 overexpression.

Twist1 is a well-known regulator of transcription during tumor initiation, stemness, angiogenesis, invasion, and metastasis [23]. Previous studies have reported elevated expression levels of Twist1 in OS tissues and that compared with non-metastatic OS (phase І/II), metastatic OS (phase III) exhibits a higher expression level of Twist1 [24]. In the present study, LAIR-1 overexpression significantly inhibited the expression level of Twist1, indicating that Twist1 is a regulator involved in LAIR-1 overexpression-mediated EMT in OS. Furthermore, Foxo1 inhibits cell migration and invasion and inhibits EMT in tumors via Twist, Snail, Slug, and zinc finger E-box-binding homeobox 1 signaling [25]. The findings of our study revealed that LAIR-1 overexpression decreased p-Akt expression at Ser473, which is a central regulator of cell survival and an upstream positive regulator of Foxo1 phosphorylation. In this study, LAIR-1 overexpression interfered with the Foxo1/Twist1 pathway, suggesting the LAIR-1/Foxo1/Twist1 feedback loop as a novel mechanism underlying OS cell migration and EMT.

Tumor cells demonstrate accelerated metabolism, high energy requirements, and increased glucose uptake. ANGPTL4 plays a key role in coordinating the increased cellular energy influx crucial for EMT. Knockdown of ANGPTL4 reportedly suppresses adenylate energy charge elevation, thus delaying EMT [26]. Upregulation of ANGPTL4 is associated with a poor prognosis of cancers and promotes tumor cell proliferation and migration, including OS cells [27, 28]. STC1, as well as STC2, was originally identified as a calcium/phosphate-regulating hormone. Recent studies have indicated that the STC-1 gene is closely related to glucose and lipid metabolism as well as to mitochondrial function [29, 30]. In addition, PARP or PAR alterations have been described in tumors, specifically those influencing EMT [31–33]. Our RNA-seq and qPCR results strongly suggest that LAIR-1 inhibits EMT through metabolic pathways.

Glucose metabolism in cancer cells contributes to their proliferation, metastasis, and therapy resistance [34, 35]. Glut1 is abundantly expressed in cancer cells, and it plays a pivotal role in glucose metabolism in tumor cells [36]. Overexpression of LAIR-1 in OS cells inhibits Glut1 expression and may further regulate glucose metabolism, thus inhibiting EMT and OS progression.

## Conclusions

The findings of our study demonstrated that LAIR-1 are overexpressed in human OS tissues and that LAIR-1 expression is significantly higher in the T2 stage OS tumor than in the T1 stage OS tumors. LAIR-1 overexpression remarkably reduces OS cell migration and inhibits EMT via Twist1 regulation. Evaluation of the underlying molecular mechanism revealed a regulatory role of LAIR-1 in Glut1-related glucose metabolism. Thus, LAIR-1 is a potential diagnostic and prognostic marker and therapeutic target for OS.

## Supplementary information

**Supplementary Fig. 1. **Effect of LAIR-1 overexpression on OS cell growth. (A) Western blotting to determine LAIR-1 expression in hFOB1.19 and OS cell lines. (B) E-cadherin and vimentin expression was analyzed by western blotting in blank, LV-NC-overexpressing, and LV-LAIR-1-overexpressing OS cells. (C) Transfection efficiency at 48 h after LAIR-1 overexpression (LV-LAIR-1) in HOS cells. Original magnification ×200. (D) LAIR-1 overexpression efficiency in HOS cells was analyzed by qPCR. GAPDH was used as an internal control. ** *P* < 0.01. (E) EdU proliferation assay analysis was performed at 48 h after LV-NC or LV-LAIR-1 lentivirus infection. Untreated HOS cells correspond to the blank group. Cell nuclei were stained with DAPI (blue). Original magnification ×200.

**Supplementary Table 1.** The RNA-seq analysis details.

**Supplementary Table 2.** Primer sequence.

## Data Availability

All data generated or analyzed during this study are included in this published article.

## Abbreviations

ANGPTL4: Angiopoietin-like 4
EMT: Epithelial–mesenchymal transition
ERK: Extracellular signal-regulated kinase
Foxo1: Forkhead box 1
GAPDH: Glyceraldehyde 3-phosphate dehydrogenase
GFP: Green fluorescent protein
Glut: Glucose transporter
IHC: Immunohistochemistry
KEGG: Kyoto Encyclopedia of Gene and Genomes
LAIR-1: Leukocyte-associated immunoglobulin-like receptor-1
LV: Lentiviral
NC: Negative control
NK: Natural killer
OS: Osteosarcoma
PARP: Poly ADP-ribose transferase
PCNA: Proliferating cell nuclear antigen
qPCR: Quantitative real-time polymerase chain reaction
RNA-seq: RNA-sequencing
siRNA: Short-interfering RNA
STC: Stanniocalcin

## References

[1] Han K, Zhou Y, Gan ZH, Qi WX, Zhang JJ, Fen T, Meng W, Jiang L, Shen Z, Min DL (2014). p21-activated kinase 7 is an oncogene in human osteosarcoma. Cell Biol Int.

[2] Dong X, Lv B, Li Y, Cheng Q, Su C, Yin G (2017). MiR-143 regulates the proliferation and migration of osteosarcoma cells through targeting MAPK7. Arch Biochem Biophys.

[3] Tetreault MP, Yang Y, Katz JP (2013). Kruppel-like factors in cancer. Nat Rev Cancer.

[4] Ma H, Su R, Feng H, Guo Y, Su G, Ma H, Su R, Feng H, Guo Y, Su G (2019). Long noncoding RNA UCA1 promotes osteosarcoma metastasis through CREB1-mediated epithelial-mesenchymal transition and activating PI3K/AKT/mTOR pathway. J Bone Oncol.

[5] Zhang L, Zhang L, Xia X, He S, He H, Zhao W (2016). Kruppel-like factor 4 promotes human osteosarcoma growth and metastasis via regulating CRYAB expression. Oncotarget.

[6] Liu Y, Zhang F, Zhang Z, Wang D, Cui B, Zeng F, Huang L, Zhang Q, Sun Q (2017). High expression levels of Cyr61 and VEGF are associated with poor prognosis in osteosarcoma. Pathol Res Pract.

[7] Xu G, Guo Y, Xu D, Wang Y, Shen Y, Wang F, Lv Y, Song F, Jiang D, Zhang Y (2017). TRIM14 regulates cell proliferation and invasion in osteosarcoma via promotion of the AKT signaling pathway. Sci Rep.

[8] Meyaard L (2008). The inhibitory collagen receptor LAIR-1 (CD305). J Leukoc Biol.

[9] Zhang Y, Sun H, Li J, Rong Q, Ji X, Li B (2017). The leukocyte-associated immunoglobulin (Ig)-like receptor-1 modulating cell apoptosis and inflammatory cytokines secretion in THP-1 cells after Helicobacter pylori infection. Microb Pathog.

[10] Wu X, Zhang L, Zhou J, Liu L, Fu Q, Fu A, Feng X, Xin R, Liu H, Gao Y (2019). Clinicopathologic significance of LAIR-1 expression in hepatocellular carcinoma. Current problems in cancer.

[11] Wang Y, Zhang X, Miao F, Cao Y, Xue J, Cao Q, Zhang X (2016). Clinical significance of leukocyte-associated immunoglobulin-like receptor-1 expression in human cervical cancer. Exp Ther Med.

[12] Cao Q, Fu A, Yang S, He X, Wang Y, Zhang X, Zhou J, Luan X, Yu W, Xue J (2015). Leukocyte-associated immunoglobulin-like receptor-1 expressed in epithelial ovarian cancer cells and involved in cell proliferation and invasion. Biochem Biophys Res Commun.

[13] Poggi A, Catellani S, Bruzzone A, Caligaris-Cappio F, Gobbi M, Zocchi MR (2008). Lack of the leukocyte-associated Ig-like receptor-1 expression in high-risk chronic lymphocytic leukaemia results in the absence of a negative signal regulating kinase activation and cell division. Leukemia.

[14] Singh M, Bhatia P, Shandilya JK, Rawat A, Varma N, Sachdeva MS, Trehan A, Bansal D, Jain R, Totadri S (2018). Low expression of leucocyte associated immunoglobulin like receptor-1 (LAIR-1/CD305) in a cohort of pediatric acute lymphoblastic leukemia cases. Asian Pac J Cancer Prev.

[15] Li W, Wei Z, Liu Y, Li H, Ren R, Tang Y (2010). Increased 18F-FDG uptake and expression of Glut1 in the EMT transformed breast cancer cells induced by TGF-beta. Neoplasma.

[16] Zhang Y, Ding Y, Huang Y, Zhang C, Jin B, Zhuang R (2013). Expression of leukocyte-associated immunoglobulin-like receptor-1 (LAIR-1) on osteoclasts and its potential role in rheumatoid arthritis. Clinics.

[17] Meyaard L, Adema GJ, Chang C, Woollatt E, Sutherland GR, Lanier LL, Phillips JH (1997). LAIR-1, a novel inhibitory receptor expressed on human mononuclear leukocytes. Immunity.

[18] Rygiel TP, Stolte EH, de Ruiter T, van de Weijer ML, Meyaard L (2011). Tumor-expressed collagens can modulate immune cell function through the inhibitory collagen receptor LAIR-1. Molecular immunology.

[19] Jiang S, Zhou F, Zhang Y, Zhou W, Zhu L, Zhang M, Luo J, Ma R, Xu X, Zhu J (2020). Identification of tumorigenicity-associated genes in osteosarcoma cell lines based on bioinformatic analysis and experimental validation. Journal of Cancer.

[20] Yu L, Liu S, Guo W, Zhang C, Zhang B, Yan H, Wu Z (2014). hTERT promoter activity identifies osteosarcoma cells with increased EMT characteristics. Oncol Lett.

[21] Poudel B, Kim DK, Ki HH, Kwon YB, Lee YM, Kim DK (2014). Downregulation of ERK signaling impairs U2OS osteosarcoma cell migration in collagen matrix by suppressing MMP9 production. Oncol Lett.

[22] Biswas KH (2020). Molecular mobility-mediated regulation of E-cadherin adhesion. Trends in biochemical sciences.

[23] Zhao Z, Rahman MA, Chen ZG, Shin DM (2017). Multiple biological functions of Twist1 in various cancers. Oncotarget.

[24] Lei P, Ding D, Xie J, Wang L, Liao Q, Hu Y (2015). Expression profile of Twist, vascular endothelial growth factor and CD34 in patients with different phases of osteosarcoma. Oncol Lett.

[25] Dong T, Zhang Y, Chen Y, Liu P, An T, Zhang J, Yang H, Zhu W, Yang X (2017). FOXO1 inhibits the invasion and metastasis of hepatocellular carcinoma by reversing ZEB2-induced epithelial-mesenchymal transition. Oncotarget.

[26] Teo Z, Sng MK, Chan JSK, Lim MMK, Li Y, Li L, Phua T, Lee JYH, Tan ZW, Zhu P (2017). Elevation of adenylate energy charge by angiopoietin-like 4 enhances epithelial-mesenchymal transition by inducing 14-3-3gamma expression. Oncogene.

[27] Nie D, Zheng Q, Liu L, Mao X, Li Z (2019). Up-regulated of angiopoietin-like protein 4 predicts poor prognosis in cervical cancer. Journal of Cancer.

[28] Zhang T, Kastrenopoulou A, Larrouture Q, Athanasou NA, Knowles HJ (2018). Angiopoietin-like 4 promotes osteosarcoma cell proliferation and migration and stimulates osteoclastogenesis. BMC cancer.

[29] Yang K, Yang Y, Qi C, Ju H (2019). Effects of porcine STC-1 on cell metabolism and mitochondrial function. General and comparative endocrinology.

[30] Sarapio E, De Souza SK, Model JFA, Trapp M, Da Silva RSM (2019). Stanniocalcin-1 and -2 effects on glucose and lipid metabolism in white adipose tissue from fed and fasted rats. Canadian journal of physiology and pharmacology.

[31] Schacke M, Kumar J, Colwell N, Hermanson K, Folle GA, Nechaev S, Dhasarathy A, Lafon-Hughes L: PARP-1/2 inhibitor olaparib prevents or partially reverts EMT induced by TGF-beta in NMuMG cells. *International journal of molecular sciences* 2019, **20**(3). 10.3390/ijms20030518 PMC6387051 30691122

[32] Rodriguez MI, Gonzalez-Flores A, Dantzer F, Collard J, de Herreros AG, Oliver FJ (2011). Poly(ADP-ribose)-dependent regulation of Snail1 protein stability. Oncogene.

[33] Masutani M, Fujimori H (2013). Poly(ADP-ribosyl)ation in carcinogenesis. Molecular aspects of medicine.

[34] Shibuya K, Okada M, Suzuki S, Seino M, Seino S, Takeda H, Kitanaka C (2015). Targeting the facilitative glucose transporter GLUT1 inhibits the self-renewal and tumor-initiating capacity of cancer stem cells. Oncotarget.

[35] Jang M, Kim SS, Lee J (2013). Cancer cell metabolism: implications for therapeutic targets. Experimental & molecular medicine.

[36] Szablewski L (2013). Expression of glucose transporters in cancers. Biochimica et biophysica acta.

