# Supplementary figures and images for: LAIR-1 overexpression inhibits epithelial–mesenchymal transition in osteosarcoma via GLUT1-related energy metabolism
Source: World J Surg Oncol. 2020 Jun 20;18:136. doi: 10.1186/s12957-020-01896-7 (PMC7345510; doi:10.1186/s12957-020-01896-7)

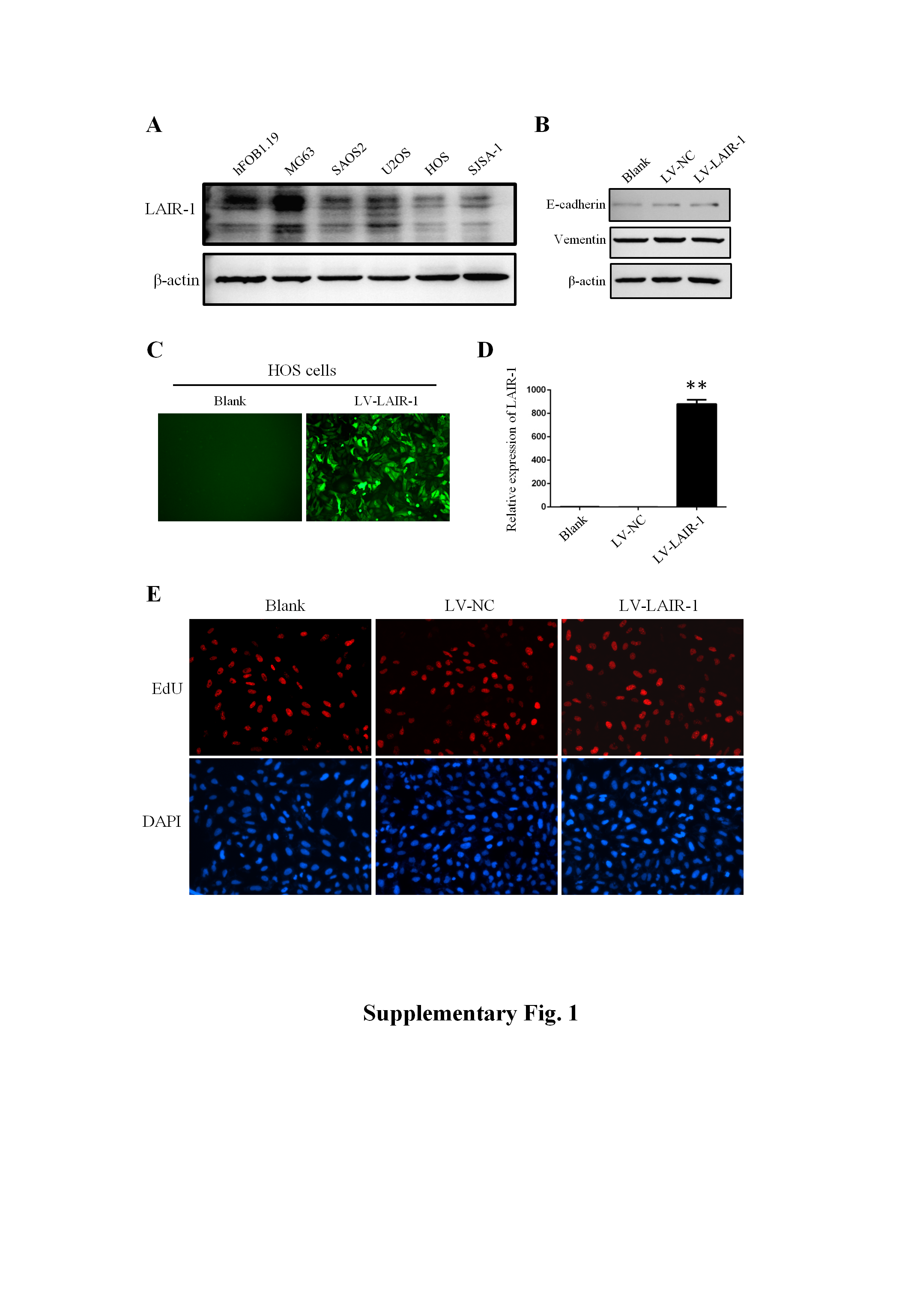

Supplement: Supplementary file 1 — Supplementary Fig. 1. Effect of LAIR-1 overexpression on OS cell growth. (A) Western blotting to determine LAIR-1 expression in hFOB1.19 and OS cell lines. (B) E-cadherin and vimentin expression was analyzed by western blotting in blank, LV-NC-overexpressing, and LV-LAIR-1-overexpressing OS cells. (C) Transfection efficiency at 48 h after LAIR-1 overexpression (LV-LAIR-1) in HOS cells. Original magnification ×200. (D) LAIR-1 overexpression efficiency in HOS cells was analyzed by qPCR. GAPDH was used as an internal control. ** P < 0.01. (E) EdU proliferation assay analysis was performed at 48 h after LV-NC or LV-LAIR-1 lentivirus infection. Untreated HOS cells correspond to the blank group. Cell nuclei were stained with DAPI (blue). Original magnification ×200. [file 12957_2020_1896_MOESM1_ESM.tif]
