# Supplementary material for: LAIR-1 overexpression inhibits epithelial–mesenchymal transition in osteosarcoma via GLUT1-related energy metabolism
Source: World J Surg Oncol. 2020 Jun 20;18:136. doi: 10.1186/s12957-020-01896-7 (PMC7345510; doi:10.1186/s12957-020-01896-7)
Supplement: Supplementary file 3 — Supplementary Table 2. Primer sequence. [file 12957_2020_1896_MOESM3_ESM.docx]

Primer sequence

| gene | Forword (5’-3’) | Reserve (5’-3’) |
| --- | --- | --- |
| GAPDH | CCGGGAAACTGTGGCGTGATGG | AGGTGGAGGAGTGGGTGTCGCTGTT |
| LAIR-1 | GGCTGTGACCGGAACTGTG | TTCTGTGCCTGTTTTAACCCAA |
| E-cadherin | ATGGCTTCCCTCTTTCATCTCCTG | TTCATAGTTCCGCTCTGTCTTTGG |
| N-cadherin | GGTGGAGGAGAAGAAGACCAG | GGCATCAGGCTCCACAGT |
| Vimentin | TCAGAATATGAAGGAGGAAATGGC | TCAGGGAGGAAAAGTTTGGAAGAG |
| Twist1 | CCTCGGACAAGCTGAGCAAGAT | CCCCACGTCGCCGCGCCAGGAATG |
| Angptl4 | AAGCCTGCCCGAAGAAAGAG | ACTGTCCAGCCTCCATCTGA |
| Stc1 | ATTCCCACCAACAAAATCCA | GGAAAAACATGGCAGAGGAA |
| Parp2 | GCCTTGCTGTTAAAGGGCAAA | TCCTTCACACTCCACATGAGCC |
